# Obstruction-Aware Signal-Loss-Tolerant Indoor Positioning Using Bluetooth Low Energy

**DOI:** 10.3390/s21030971

**Published:** 2021-02-01

**Authors:** Aybars Kerem Taşkan, Hande Alemdar

**Affiliations:** Department of Computer Engineering, Middle East Technical University, Ankara 06800, Turkey; aybars.taskan@metu.edu.tr

**Keywords:** indoor positioning, Bluetooth Low Energy, particle filter, multilateration

## Abstract

Indoor positioning is getting increased attention due to the availability of larger and more sophisticated indoor environments. Wireless technologies like Bluetooth Low Energy (BLE) may provide inexpensive solutions. In this paper, we propose obstruction-aware signal-loss-tolerant indoor positioning (OASLTIP), a cost-effective BLE-based indoor positioning algorithm. OASLTIP uses a combination of techniques together to provide optimum tracking performance by taking into account the obstructions in the environment, and also, it can handle a loss of signal. We use running average filtering to smooth the received signal data, multilateration to find the measured position of the tag, and particle filtering to track the tag for better performance. We also propose an optional receiver placement method and provide the option to use fingerprinting together with OASLTIP. Moreover, we give insights about BLE signal strengths in different conditions to help with understanding the effects of some environmental conditions on BLE signals. We performed extensive experiments for evaluation of the OASLTool we developed. Additionally, we evaluated the performance of the system both in a simulated environment and in real-world conditions. In a highly crowded and occluded office environment, our system achieved 2.29 m average error, with three receivers. When simulated in OASLTool, the same setup yielded an error of 2.58 m.

## 1. Introduction

Indoor positioning systems (IPSs) are useful, especially in large environments such as big malls, stores, museums and similar places [1,2]. In those environments, what to do and where to go without spending much time on looking for directions can be very difficult. For the visually impaired, the handicapped and the elderly, indoor navigation can be more difficult [3,4]. Navigation systems that direct people can make use of IPS to work effectively. Besides, IPSs provide many business and marketing benefits. For instance, a store owner may wonder where their customers spend time and what their dwell times in the store are. A museum application can send informative push notifications to the visitors when they look at a particular painting or an antique piece by knowing their positions.

There are two main approaches to indoor localization, namely, infrastructure-based and infrastructure-less approaches [5]. In the latter, the fingerprints from the ambient features such as light, sound, magnetic signals, or smartphone sensors are used in general [6]. Infrastructure-based methods can employ pre-installed video-cameras or wireless technologies. such as Wi-Fi, ZigBee, ultra wide band (UWB), radio frequency identification (RFID) and frequency modulation (RF) radio and Bluetooth Low Energy (BLE). Infrastructure-based IPSs can be expensive either due to the methods used or due to expensive hardware components. For example, using video camera technologies may not be preferable due to expensive hardware cost associated.

Wireless signal technologies for indoor positioning are on average less expensive than the video-based alternatives. Some wireless technologies could be more preferable than others in terms of cost. For example, Bluetooth Low Energy (BLE) gives broader opportunities when compared to other wireless methods. BLE is a common wireless technology that is generally used in close proximity, and is supported by Android, iPhone operating system (iOS) and many other devices. BLE uses very low energy which means long battery life for the device having BLE. While ZigBee consumes low energy as BLE does, ZigBee is not as widely supported. Wi-Fi is widely supported, yet it has a higher energy requirement than BLE.

Solutions using BLE may become expensive in some cases due to expensive hardware requirements such as high quality antennas; using many transmitters called the beacons; using many receivers; or using beacons with battery-draining parameters such as low advertisement interval and high transmission power [7]. However, the costs can be optimized with the use of intelligent and robust algorithms that can function with less requirements. In this paper, we propose such a method, providing a cost-effective solution to the indoor location tracking problem using BLE. Our proposed approach can tolerate the signal-losses, leaving the fingerprinting as an optional step. With these features, it can work with minimal setup without the need for expensive antennas or extra beacons for fingerprinting. However, it provides the flexibility of working with fingerprinting or having more receivers.

We present a novel wireless indoor positioning algorithm called obstruction-aware signal-loss-tolerant indoor positioning (OASLTIP) that has a unique pipeline for BLE-based indoor tracking that consists of pre-filtering, multilateration and particle filtering-based tracking. Since we offer an infrastructure-based solution, the receiver placement should be completed before the beginning of continuous operation. After the installation, if preferred, fingerprinting beacons can also be deployed, but they are not required. The receiver positions, obstruction locations and their material types and widths are provided to our algorithm. OASLTIP method tracks the position of a BLE tag by using the signals received from the tag. In order to alleviate the effects of the inherent noise, we prefilter and smooth the signals using a running average filter and threshold. After that, the obstruction-aware multilateration algorithm is applied to measure the most probable location of the tag. In order to improve the prediction accuracy, we also use particle filtering so that we incorporate the previous movements of the tag into our prediction, having a more consistent prediction path for the tracked tag. At each stage of the pipeline, we have the following contributions.

- In the prefiltering step, we not only smooth the signal with running average filtering, but also decide on its usability. We do not let pass the low-quality measurements that can reduce the tracking performance.
- In the multilateration stage, we can tolerate the loss of signal measurement by imputing it with the information from the previous time-steps.
- We consider the architectural constraints and the obstructions in the environment. We consider the signal propagation losses due to the obstructions by approximating the loss using the thickness and type of material that is on the way between the receiver and the beacon. Our method is unique in the way it handles the obstructions in the indoor environment.
- We do not require the use of fingerprinting beacons, and we do not restrict them. Fingerprinting is an optional step that can be used in multilateration part.
- We provide an additional tracking algorithm to get a better and more smoothly estimate of the location rather than using the estimate provided by the multilateration only. This allows us to consider the previous movement of the tracked tag and providing a more consistent estimate for the location of the tag.

In addition to these methodological contributions, we are also releasing our simulation tool not only to reproduce our results but also to simulate, visualize and report results for other scenarios that are configured easily. We employed a parametrized and flexible approach so that our solution is applicable and can be tested for different conditions. We performed several experiments to validate our approach in challenging conditions both in simulated and in real-world environments. We also validated our simulated environment’s performance by generating the real-world setup in our flexible simulation tool and repeating the real-world experiments there. In this way, we have more insights into the approximation capability of our simulation tool when compared to real-world scenarios. To the best of our knowledge, this is the only study that has taken such an approach. Finally, we provide our insights about BLE signals’ behavior in terms of different chip sets, different carrying positions, different transmission powers and different obstruction materials.

The rest of this paper is as organized as follows. We survey several studies in the literature together with a comparison summary table in Section 2. In Section 3, we describe the details of our proposed algorithm for indoor positioning. Section 4 describes the details of the simulation tool that is used for the experimental evaluation, which is detailed in Section 5. Finally, we conclude with Section 6.

## 2. Related Work

Indoor positioning can be done using different wireless signal technologies, such as Wi-Fi, ZigBee, ultra wide band (UWB), radio frequency identification (RFID), frequency modulation (RF) radio and Bluetooth Low Energy (BLE) [5]. In this study, we use BLE technology for indoor environment tracking. We cite several related works on the subject and explain how our approach differs from the other approaches or how we contribute to these approaches. We explain the environmental setup, applied methods and accuracy of these studies.

Subhan et al. [8] used linear discriminant analysis (LDA) to predict location dynamically based on RSSI signals in an indoor environment of 10 m × 10 m. They used predefined trajectories for evaluation purposes and reported an average of 79% accuracy. Giuliano et al. [2] used four receivers in a 14 m × 12 m area to perform localization. They considered obstructions due to humans but no architectural obstruction was used. They used a neural network approach to convert RSSI valuers to distances; we used the path loss formulas for that purpose. They reported error values around 1 m to 6 m depending on the scenario.

Khan [9] and Nguyen et al. [10] applied trilateration with a few other methods for positioning. Khan [9] did a comparative study of existing indoor positioning approaches, such as the least squares-based approach and min-max based positioning algorithms. He stated that the min-max based approach is better for accuracy and it is low cost. The min-max algorithm functions by constructing a bounding box for each anchor node based on the distance. Then three boxes are constructed according to distances and the intersection of these boxes determines the resulting position. Nguyen et al. [10] used several filtering methods for preprocessing the RSSI values they collected; some of these filtering methods used were the Gaussian filter, a Kalman filter and simply averaging the RSSI values collected. They used an improved least squares method for positioning and compared this method with other techniques, such as least square estimation (LSE). The authors got an error of 0.192 m using their improved LSE methods; classic LSE methods resulted in 0.333 m.

Kalbandhe and Patil [11] indicated that positioning with BLE is more accurate than positioning with Wi-Fi. They used mobile phones as the receiver devices. They used two different beacons and obtained fourteen distance vs. RSSI results for both of the beacons; all of the distances used in measurements were between 0 m and 3 m. The authors indicated that they were able to classify signal sources correctly for near, immediate and far positions; those positions represent 0–0.5 m, 0.5–3 m and above m respectively.

Kriz et al. [12], Teran and Carrillo [13], Li and Ma [14] and Huang et al. [15] followed a hybrid approach using BLE and Wi-Fi. Kriz et al. used four Wi-Fi access points and got 1 m error on average, and up to seventeen BLE beacons decreased the error from 1 m to 0.77 m in an area of 52 m × 43 m. Teran and Carrillo [13] used a hybrid system for wireless local area network (WLAN) and BLE positioning using ML cloud services. The authors compared the hybrid method with Wi-Fi only and BLE only. For positioning, SVM and KNN classifiers were used. They got better accuracies using Wi-Fi and BLE together, 75% of the time, for sub-meter ranges using KNN. However, using only BLE, they get a sub-meter accuracy, 68% of the time using KNN and 60% of the time using SVM. Li and Ma [14], used BLE tags and BLE/Wi-Fi repeaters. Their system does positioning calculations using two values: RSSI fingerprint and cell of origin (CoO). They performed experiments in a rest area and an office area and got errors of 1.2 m and 1.37 m for these areas respectively. Huang et al. [15] tried to come up with a solution for environments where a lot of Bluetooth sources exist; these Bluetooth sources could be Bluetooth classic devices instead of BLE. Since other Bluetooth devices would cause interference, they overcame the interference with their methods. They call their method a hybrid due to fusing dead reckoning and trilateration methods. The authors applied three methods: trilateration, dead reckoning and the hybrid method. Trilateration, dead reckoning and hybrid methods had root mean squared errors (RMSE) of 2.33, 0.82 and 0.76 m respectively. Thus, the hybrid method outperformed the other two methods.

Memon et al. [16] implemented a mobile indoor positioning system to track staff members of a university department. The authors did the experiments in a laboratory environment and split the area into blocks. The accuracy in terms of blocks was 94%.

Sie and Kuo [17] used BLE beacons with a transmission period of 100 ms. The authors performed different experiments with two TX power levels, −30 and −42 dBm, and collected RSSI values every 50 cm. According to results, −42 dBm can only be used within 0–50 cm and with −30 dBm; they got errors up to 1.5 m.

Radoi et al. [18] performed some experiments in an office environment. The authors analyzed two methods: fingerprinting and particle filtering. For a room of size 8 m × 6 m, for errors lower than 2 m, the fingerprinting method performed better. For errors higher than 2 m, the particle filter performed better than fingerprinting. Particle filtering gave an error of 4 m at maximum, 97.5% of the time. Malekzadeh et al. [19] proposed improving the particle filtering-based approach using a boxing scheme. However, their method requires the use of fingerprinting beacons.

Teran et al. [20] implemented a solution based on Internet of Things (IoT). The experimentation area was 5 × 5 = 25 m^2^ which was further divided by 1 × 1 m^2^ blocks for accuracy estimations. They got an error of 2 m using two machine learning classifiers.

Paterna et al. [21] used different channels of BLE technology to lower the effects of signal degrading phenomena such as reflection, refraction and loss of BLE signals due to external objects or signals. The authors did the experiments in three different areas with sizes 4.8 m × 6 m, 9.19 m × 6.18 m and 17.6 m × 16.5 m, and got errors of 2, 1.82 and 4.6 m with a 90% confidence interval. Kajioka et al. [22] reported a 0.8 m error which was obtained in an indoor environment of size 10.5 m × 15.6 m.

Zue et al. [23] proposed a graph optimization-based approach which combines fingerprinting-based methods and range-based methods. The authors performed a test in an area of 90 m × 37 m with two different numbers of beacons, 24 beacons (sparse) and 48 beacons (dense), to see the effect of beacon density on positioning estimations. They got errors of 2.26 and 1.27 m with the sparse beacon environment and dense beacon environment, respectively.

Mekki et al., [24] implemented an Internet of Things (IoT)-based solution. The authors gave some path loss exponents for different environments, such as free space and obstructions in buildings and factories, since in some environments signal strength loss is higher than the others. They got an error of 1.5 m.

In Table 1, we summarize the related indoor positioning studies that used Bluetooth Low Energy (BLE) signals by giving information about the performance, the test area size (width × height) and the methods used. Although the studies are not directly comparable, the table is still sorted by the reported MAE of the studies. We report our real-world experimental results in the table, which places this work in the middle range. In Table 1, the hardware that the other studies used is placed in the column named "Deployment Requirements." We can see that our solution uses less equipment than most of the other approaches, and our test area was larger than half of the other approaches. Moreover, our method is unique in the way it handles the obstructions in the indoor environment compared to the other methods.

## 3. Obstruction-Aware Signal-Loss-Tolerant Indoor Positioning (OASLTIP)

In this section, we present our method for indoor positioning using BLE technology. Our method consists of four stages. The first stage is the receiver placement followed by the optional fingerprinting stage. Most of the indoor positioning systems have this kind of installation stages before the continuous operation.

**Figure 1:**
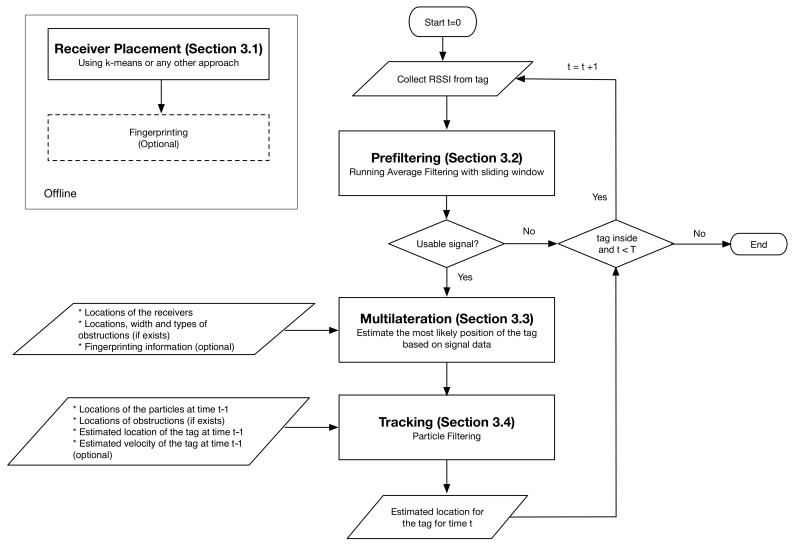
Overview and flow chart of the OASLTIP method.

After the initial setup has been completed, our OASLTIP method starts tracking the position of a BLE tag by receiving signals from it. The signals are first prefiltered to eliminate the adverse effects of the inherent noise. After that, our obstruction-aware multilateration algorithm is applied to measure the probable location of the tag and finally particle filtering is applied to reduce the variance in the final location accuracy by incorporating the historical movements of the tag. The overview of the method and the flow chart is presented in Figure 1. The method can be configured to run for a predetermined time-steps, *T*, or it can decide the presence of tag itself.

### 3.1. Receiver Placement and Fingerprinting

For optimal tracking performance with minimum cost, we have to install the receiver devices carefully given the indoor map. We need to place the least amount of receiver devices and still manage to receive signals regardless of where the tag is inside the environment. Placing lots of receiver devices does not contribute to locating the tag more accurately if all the devices are installed close to each other, they must be placed carefully. In order to address this problem, we propose a receiver positioning method for rectangular shaped indoor environments as it is the most commonly found shape for an indoor store. Besides, we can easily approximate most building shapes with a rectangle or a set of rectangles. Our method can be run multiple times to find the optimal places in each of the rectangles. For more complex shapes that cannot be approximated by a set of rectangles, human eye would be more accurate for efficient positioning. The OALSTIP method uses the locations of the receivers as input and it does not assume that they are optimal.

We model the receiver positioning problem as a clustering problem where each cluster consists of points formed around a receiver. Therefore, finding the cluster centers is equal to finding the places for receiver devices. We use k-means algorithm for finding the clusters since it is one of the simplest and popular clustering algorithms. The k-means algorithm identifies *k* cluster centers, and then allocates every data point to the nearest cluster, while keeping the clusters as small as possible. Since the problem is computationally NP-hard, iterative refinement technique is used. The algorithm starts with an initial selection of the cluster centers and the assignment is made using the current centers, i.e., each data point is assigned to the nearest cluster. Then, in the second step, the cluster centers are re-calculated according to the assignments made. The algorithm alternates between those two stages until no more change is made. This simple procedure is derived from the expectation-maximization algorithm and it is guaranteed to converge. However, the solution is not guaranteed to be the global optimum. It may get stuck at a local minimum.

In order to initialize the algorithm for *k* receivers, we create evenly-spaced initial points spread across the map. This makes the algorithm converge faster. In order to overcome the sub-optimality, we run k-means algorithm with different initial cluster centroids. Using a hundred different runs, k-means chooses the best clusters and their centers, in terms of the inertia (within-cluster sum-of-squares criterion). Finally, we use the centers of these clusters as the receiver positions.

**Figure 2:**
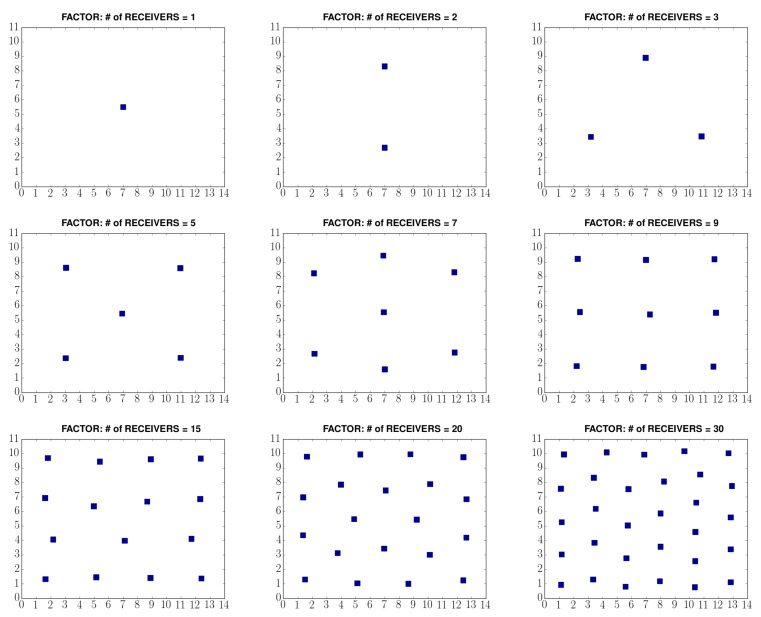
Sample output of k-means for placement of receivers.

It is important to note that the resulting receiver positions are just suggestions. There may be obstacles in the environment that prevents the installation of a receiver to a specific location. The receivers can be placed to suitable places in the environment, the tracking algorithm does not assume a specific placement for the receivers. Additionally, the placement can be done using a grid structure if the number of receivers are suitable for a grid structure. We propose using clustering algorithm since it is more convenient for some setups. In Figure 2, we show the results for several placement suggestions with different number of receivers.

After the receiver installation, optionally, we also have fingerprinting. In the experiments, we place four beacons in certain locations in the environment (which forms four reference points) as base positions to be used for interpolation and extrapolation of the map later. Then, we collect BLE signals with 10-min time interval for three days. After three days, we average all the signals caught at each four reference points. After that, using these four reference points and the distance to the receiver devices, we calculate the RSSI values at all other points in the environment where these points are separated by a certain distance. This step might be useful to mitigate the multipath effect, which is a phenomenon causing the signals reaching a receiver device via multiple paths due to signals being reflected, refracted and diffracted from the obstructions in the environment.

After the installation is finished, our system is ready to capture the signals and locate the tags roaming in the environment.

### 3.2. Prefiltering

Our OASLTIP algorithm starts with a filtering step. In this step, we prefilter the received signals to determine which signals should be let to pass for positioning calculations using running average filtering (RAF) algorithm. In RAF algorithm, we use a sliding window approach to store RSSI values. When the signal count in a sliding window reaches three with the newly arriving signal, we sort the signals by their RSSI values. Then, we determine the strongest and weakest signal and mark them as outlier signals for the current calculation. After that, we calculate the mean of the non-outlier signals in the sliding window. If the mean is greater than a predetermined RSSI threshold value, we let the newly arriving signal to be used in our further calculations. After the sliding window is full size; when a new signal arrives, we delete the oldest signal to open a place for the newly arriving signal in the sliding window. Note that, we do not use the first two signals arriving at our receiver since we need at least three signals for the mean calculation of the non-outlier signals. This step is important to use strong (reliable) BLE signals only in our positioning calculations. Empirically, we find that a window of size seven is suitable for our purposes however, this is a configurable parameter that can be set to any value greater than two.

### 3.3. Obstruction-Aware Signal-Loss-Tolerant Multilateration Algorithm

In this step, we calculate the most likely position of the tracked tag using the RSSI values at all of our receiver devices. Using the prefiltered RSSIs, the positions of the receivers, the obstruction information and a loss function, we make error minimization measurements to predict the tag location. If no RSSI value is obtained for the current time step or RSSI of the current time step gets filtered out, we look at the RSSI values at time steps which are at most some predefined number of time steps before and ahead of the current time step until we find a RSSI value. Since this is a real-time system, to obtain the next time step RSSI value, we wait and do not make any calculations for the current time step to locate the tag.

To be able to locate the tag with high accuracy, we need to know at least three distance values of the tag to three different points in the map. If the signals are not noisy, using only three reference points locates the tag with 100% correctness using an algorithm called trilateration. However, in real life, the signal values are noisy and the distance values to reference points are not known exactly. Hence, if available, using more than three reference points result in more accurate results since we would have more distance information.

For obstruction-aware signal-loss-tolerant multilateration, the values we know are the receiver positions and the RSSI values arriving at these receivers. Therefore, using RSSIs and the position of the receivers, we have to be able to predict the object location. When the tag moves, each receiver catches some signal where the RSSI value may differ in each of the receivers. Due to different RSSI values, we get different distance information to different receivers. We approximately know what the distance should be given the RSSI value. Due to the noise in the signal, in reality, the real distance values might be different from what the RSSI value tells us. Hence, we need to correct this RSSI value to extract the right distance information of the tag to the receiver device having this RSSI.

In order to find the most likely position given the prefiltered RSSIs, we form a 2-dimensional (2D) grid structure in the environment using a configurable resolution parameter, *r*, and we use a function that calculates the likelihood of presence in each discrete point in the grid.

For each 2D point $P_{j}$ in the grid, we measure the likelihood of the tag being in that location by both using the prefiltered RSSI signals and the obstructions that are present in the environment. If there is an obstruction between point $P_{j}$ and a receiver, we correct the RSSI value by how much RSSI is lost due to this obstruction. In order to approximate the loss incurred due to obstructions, we consider the type of the obstruction material and the thickness. We assume a linear signal path from point $P_{j}$ to the receiver location $R_{i}$ and construct an imaginary line segment. Then, we take the intersection amount between this line segment and the obstruction as the thickness of the obstruction we intersect and calculate the signal loss due to this obstruction accordingly. We make this calculation for each obstruction in the way of $P_{j}$ and $R_{i}$.

For each time step, we get a RSSI value at each receiver. After applying prefiltering and postfiltering on this RSSI, we convert the RSSI value to a distance value, $d{\left(R_{i},P_{t}\right)}_{RSSI}$. $R_{i}$ is the location of the receiver *i* (which is known) and $P_{t}$ is the location of the tag (which is unknown at this stage). In order to convert a given RSSI in dBm to the corresponding distance *d* in meters, we use the following equation [25].

(1) $$d=\left\{\begin{array}{cc} 10^{\left(\left(RSSI-RSSI_{1m}\right)/-20\right)} & RSSI\leq RSSI_{1m}\\ 10^{\left(\left(\left(RSSI-TX\_Power\right)*\log2\right)/\left(RSSI_{1m}-TX\_Power\right)\right)}-1 & RSSI>RSSI_{1m} \end{array}\right.$$

Moreover, we know the fingerprinting RSSI on every point in the grid if we created the fingerprinting map before the multilateration algorithm. We can calculate the distance, $d{\left(R_{i},P_{j}\right)}_{FP}$, for every point $P_{j}$ in the grid using this fingerprinting RSSI in the same way. Finally, we calculate the Euclidian distance, $d(R_{i},P_{j})$, between each point $P_{j}$ in our grid and every receiver $R_{i}$.

In our multilateration algorithm, for our measured location for the tag, we choose the grid point that minimizes the differences between $d(R_{i},P_{j})$ and/or $d{\left(R_{i},P_{j}\right)}_{FP}$, and $d{\left(R_{i},P_{t}\right)}_{RSSI}$. In order to make this minimization we also use a punishment term for weighting the differences. We have two cases depending on the signal receiving situation of the receivers.

1. **Receiver catches a signal for the current time step**
  If $d{\left(R_{i},P_{t}\right)}_{RSSI}$ is small, then the tag must be close to the receiver since strong signals generally convey right information about the distance. Hence, we expect the $d(R_{i},P_{j})$ to be small as well if we are to choose $P_{j}$ as the predicted position over the other checkpoints in multilateration.
  If $d{\left(R_{i},P_{t}\right)}_{RSSI}$ is large, it can be either since the tag is distant to the receiver or there is an obstruction between the receiver and the tag causing RSSI value to be low. Since we do not rely on low RSSI signals, we do not punish as much as we do when $d{\left(R_{i},P_{t}\right)}_{RSSI}$ is small.
  The punishment in this case is proportional to the absolute difference between $d{\left(R_{i},P_{t}\right)}_{RSSI}$ and $d(R_{i},P_{j})$, however, disproportional to $d{\left(R_{i},P_{t}\right)}_{RSSI}$. Being disproportional to $d{\left(R_{i},P_{t}\right)}_{RSSI}$ is what provides punishing more when $d{\left(R_{i},P_{t}\right)}_{RSSI}$ is small than it is large. Lastly, we square the punishment term so that we punish large errors more.
  If we want to use the fingerprinting results in our minimization algorithm, we can replace $d(R_{i},P_{j})$ with $d{\left(R_{i},P_{j}\right)}_{FP}$ for this case. However, we only punish according to fingerprinting data if prefiltered RSSI value and fingerprinting RSSI value differ more than a threshold we determine.
2. **Receiver does not catch a signal for the current time step**
  If we have no signal value to make a calculation, the tag is most probably not close to the receiver. However, we know our lowest signal threshold, and hence the maximum distance coverage, $d_{M}$, of our receiver. If no signal is received at our receiver, the probability of the tag being more distant to the receiver than $d_{M}$ meters is more than the tag being in this coverage. Therefore, we punish the point $P_{j}$ if it is in the coverage area of the receiver. The punishment is disproportional to the $d(R_{i},P_{j})$ since the point $P_{j}$ should be as distant to the receiver as possible when in coverage area. Lastly, we take the square of the punishment term so that we punish large errors more. To use the fingerprinting results in our minimization algorithm, we can replace each $d(R_{i},P_{j})$ parameter with $d{\left(R_{i},P_{j}\right)}_{FP}$ for this case.

### 3.4. Tracking and Localization

The multilateration algorithm outputs a location for the tag using the signal levels. We can use this prediction as the tag location, however, with the help of tracking, we can more precisely localize the tag in the environment since we also take into account the previous location, direction and velocity of the tag into account.

In order to locate and track the BLE tag, we use nonlinear Bayesian filtering approach, in which we estimate the state of the dynamical system from sensor measurements using a probabilistic Bayesian approach. Unlike its linear counterparts like Kalman filter [26], a nonlinear Bayesian approach like the particle filter we use, can handle more complex motion patterns that are not linear. Additionally, it does not make Gaussian noise assumptions and can handle multiple targets.

When we have a probabilistic model on how the system changes in response to inputs received in time, and a model of what observations we would expect to see in particular states, we can use Bayesian filter to track the state of the system. In our case, the state of the system represents the location of the tag and the observations are the measured RSSI values obtained from the receivers. In nonlinear Bayesian filtering, we have two models, i.e., the system model and the measurement model. We represent these models as probabilistic functions and apply the Bayes theorem to find the posterior probability density function (pdf) of the state based on the available measurements. Such a filtering mechanism is composed of two steps.

- Prediction: The system model is used to predict the state pdf before the actual measurement is made. The resulting prediction is noisy and the uncertainty is high.
- Update: We use the latest measurement to modify the prediction pdf of the state and obtain the posterior pdf using the Bayes theorem.

The tracking algorithm is the continuous application of these two consecutive steps. For most of the time, however, the posterior pdf cannot be analytically calculated in closed form. That is why, approximate solutions are used. Particle filtering is one of the approximation techniques that is widely used. The main idea is to approximate the full posterior pdf with a weighted set of samples called the particles and the sequential importance resampling technique is used to update the weights at each iteration.

There are four stages in the tracking method: initialization, prediction, update and resampling. Initialization is done only once. Resampling is done when necessary. Prediction and update step are done at each iteration. At each iteration, we can make the localization for the tag after the update step.

#### 3.4.1. Initialization

We initialize the tracking algorithm by randomly placing *N* equally weighted particles across the map of the environment. This step is only done once at the beginning, t=0. We denote the set of particles as for time *t* ad $P_{t}=\left\{\left[x_{t}^{i},y_{t}^{i}\right],w_{t}^{i}:i=1,\ldots ,N\right\}$, where $\left[x_{t}^{i},y_{t}^{i}\right]$ denotes the *x* and *y* coordinates of the particle *i* at time *t* and $w_{t}^{i}$ is the weight of the particle for this time-step. at the initialization phase $w_{0}^{i}=1,\;\forall i$.

#### 3.4.2. Prediction

We predict the position of the object using the system model. Since we track humans in an indoor environment, we use a motion model suitable for human movement for this step. In our motion model, we define a maximum limit for displacement, $\Delta_{M}$, which represents the maximum number of meters a person move in one time step. Additionally, we consider the speed of the human with a configurable weighting factor $c_{\upsilon }$

(2) $$p\left(\left[x_{t},y_{t}\right]|\left[x_{t-1},y_{t-1}\right]\right)=\left(1-c_{\upsilon }\right)\left[x_{t-1}+U\left(-\Delta_{M},\Delta_{M}\right),y_{t-1}+U\left(-\Delta_{M},\Delta_{M}\right)\right]+c_{\upsilon }\upsilon_{t-1}$$

where $\upsilon_{t-1}=\left|\right|\left[x_{t-1},y_{t-1}\right]-\left[x_{t-2},y_{t-2}\right]{\left|\right|}^{2}$ and U(a,b) is the uniform distribution with parameters *a* and *b*.

The speed at the previous time-step, $\upsilon_{t-1}$, gives idea about how the tag will move in this iteration since humans would make a movement with a similar speed most of the time. If the speed weighting factor, $c_{\upsilon }$ is close to 1, we guess that the current movement will be very similar to the previous one. If it is closer to 0, we consider the previous velocity less. This flexible approach makes the motion model flexible for different scenarios.

In the prediction step of the particle filter tracking algorithm, every particle moves according to the probabilistic system model given in Equation (3). The position of each particle *i* is calculated as follows:

(3) $$\left[x_{t}^{i},y_{t}^{i}\right]=\left(1-c_{\upsilon }\right)\left[x_{t-1}^{i}+U\left(-\Delta_{M},\Delta_{M}\right),y_{t-1}^{i}+U\left(-\Delta_{M},\Delta_{M}\right)\right]+c_{\upsilon }\upsilon_{t-1}^{i}$$

#### 3.4.3. Update

In the prediction step of the tracking algorithm, every particle moves probabilistically according to the model and no measurement data is used. In the update step, the measurement data is used to update the weights of the particles. We update the particle weights proportional to the distances between particle positions and measured position of the multilateration algorithm. We give higher weights to the particles which are closer to the measured location of the tag.

(4) $$w_{t}^{i}\propto w_{t-1}^{i}d\left(\left[x_{t}^{i},y_{t}^{i}\right],\left[x_{t}^{*},y_{t}^{*}\right]\right)$$

where d(a,b) represents the Euclidian distance between points *a* and *b* and $\left[x_{t}^{*},y_{t}^{*}\right]$ is the measured location of the tracked tag at time *t*.

#### 3.4.4. Resampling

Particle filter algorithm suffers from a particle degeneracy problem due to the weight update. As algorithm iterates, some of the particles’ contribution diminishes since their weights become practically zero. In other words, the degeneracy causes some of the particles becoming really heavy and bulky so that they lose the agility to track the tag effectively. Resampling is the process of sampling as many particles as we have initially. In this step, we check whether the weights of our particles are good representatives for our tracked object using an effective number of particles, $N_{eff}$, value.

If $N_{eff}$ is low, it means that there are low number of particles whose weights are large and large number of particles whose weights are small (close to 0). Hence, we need to resample all of the particles when $N_{eff}$ is lower than a threshold we determine. The threshold for resampling is generally chosen as half of the number of particles, *N*. In this study, we resample if $N_{eff}<N/2$. We calculate $N_{eff}$ follows:

(5) $$N_{eff}=\frac{1}{\sum_{i=1}^{N}{\left(w^{i}\right)}^{2}}$$

#### 3.4.5. Localization

At each iteration, the localization of the tag is achieved by predicting the location of the tag with a weighted average of all the particles. The predicted location of the tag, [${\hat{x}}_{t},{\hat{y}}_{t}]$, is calculated as

(6) $$\left[{\hat{x}}_{t},{\hat{y}}_{t}\right]=\sum_{i}w_{t}^{i}\left[x_{t}^{i},y_{t}^{i}\right]$$

Additionally, the uncertainty associated with the current prediction can be calculated with the covariance matrix of the particles and it can be visualized using the error ellipse. The error ellipse is a standard way of representing the distributions. We use it to visually display the uncertainty associated with the current location estimate, which is defined as the weighted average of particles as given in Equation (6). Since we, have two dimensions, the covariance matrix, *C* is a 2×2 matrix.

(7) $$C=\left[\begin{array}{cc} a & b\\ c & d \end{array}\right]$$

**Figure 3:**
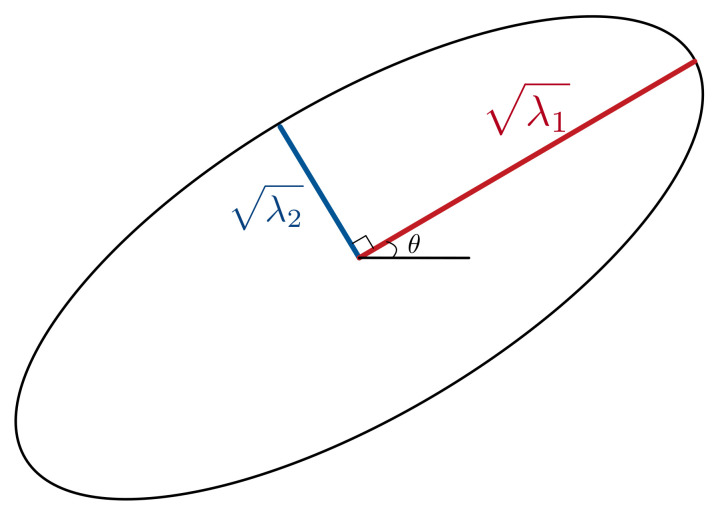
Error ellipse representing the covariance matrix.

$\lambda_{1}$ and $\lambda_{2}$ are the eigenvalues of *C*. Figure 3 shows a representative error ellipse, θ is the counter clock-wise angle between the positive x-axis and the eigenvector corresponding to the highest eigenvalue, $\sqrt{\lambda_{1}}$ is the radius of the major axis (the longer radius) and $\sqrt{\lambda_{2}}$ is the radius of the minor axis (shorter radius). When the error ellipse is wide, the particles are more disperse and our current estimate has higher uncertainty. In other words, the standard deviation of the particles is higher. It shrinks as our tracking algorithm has more signals and more observations about the tag location, having a more accurate estimate with higher confidence.

## 4. OASLT Simulation Tool

In order to validate our approach and visualize the results, we develop a simulation tool with experimentation and visualization capabilities. The OASLTool can also be used for evaluating several alternative deployment options and scenarios before the actual deployment in a real environment. We also make the source code of OASLTool publicly available (https://github.com/aybarskerem/OASLTIP).

OASLTool enables users to construct a virtual environment with architectural constraints and obstructions. The receiver placement can be done automatically using our proposed approach. Alternatively, users can place the receivers according to their own preferences.

**Figure 4:**
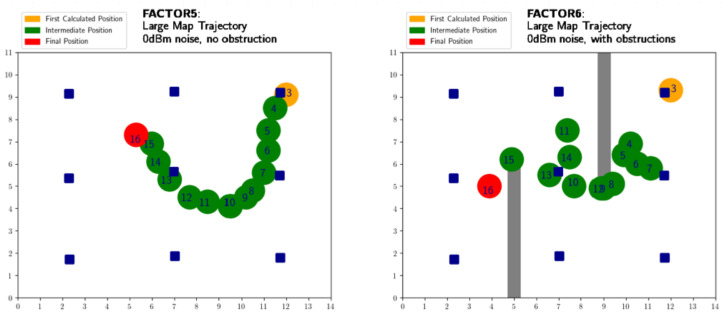
The OASLTool displays the trajectory, obstructions and placement of the receivers in the environment. Blue squares show the placement of receivers; the orange circle is the first predicted position of the tag; green circles with numbers show the intermediate time-steps; the red circle marks the end of trajectory. Gray blocks represent the concrete obstructions in the environment, such as walls. The axes show the dimensions of the environment in meters.

The OASLTIP algorithm is presented in a single Python script in OASLTool. It uses standard Python libraries like numpy, matplotlib. There are also utility scripts both in Python and Bash to plot the figures and automatize running multiple scenarios of OASLTIP with different parameters which is included in the repository besides the main script. OASLTool currently supports single floor building structures and only works for indoor environments with local coordinates. Using the provided tool, it is possible to create random movement patterns for several simulated human subjects. Both the trajectory of the target and the predictions will be displayed on the graphical user interface while collecting the experiment results. A sample graphical user interface (GUI) example is provided in Figure 4.

**Figure 5:**
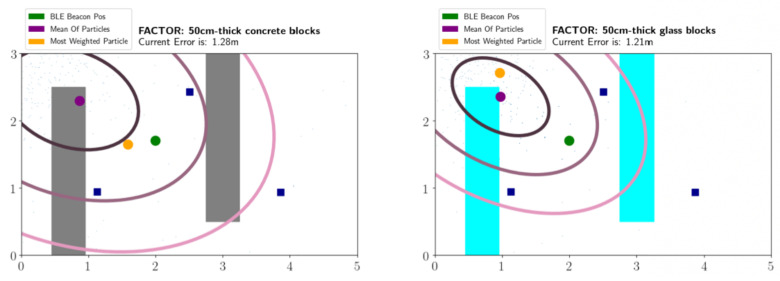
The OASLTool displays the particle filter tracking results in an animated manner. Blue squares show the placement of receivers; the green circle shows the actual position of the tag; the orange circle is the particle with the maximum weight; the purple circle is the weighted mean of the particles, which is used as the predicted position. The ellipses visualize the covariance of the particles.

On this visualized environment, we also show some outputs of the particle filtering part of the OASLTIP which are the maximum weighted particle, the mean of the all particles and three ellipses formed around the mean of the particles to represent the most likely areas that the tag is in. The innermost ellipse represents the most likely area. While we go from the innermost ellipse to the outermost ellipse, the likelihood of the tag being in the corresponding positions decrease. Real position of the tag (ground truth position), the maximum weighted particle and the mean of the particles are represented by green, orange and purple discs respectively. Blocks and rooms are represented by filled and unfilled rectangles respectively. Blocks are either shown as gray, aqua or beige colored to represent concrete, glass and plastic materials respectively whereas rooms are either shown as gray or aqua colored. Moreover, the receiver devices are shown as blue filled squares, as depicted in Figure 5.

For our experiments, we can define the following parameters in the OASLTool that can be configured.

**minUsefulSignal:** This parameter represents the minimum RSSI value to be used for our particle filtering algorithm calculations.

**minSignalValue:** This parameter represents the minimum RSSI value that can be caught by a receiver device. We do not prefilter RSSI values lower than *minSignalValue* and directly reject it since we do not expect such a low signal to be transmitted from our signal transmitting devices (beacons). *minSignalValue* is always less than or equal to *minUsefulSignal*.

**maxSignalError:** This parameter represents the maximum signal noise possible. Hence, the signal noise in the indoor environment for algorithm is always between 0 dBm and *maxSignalError*.

**sensitivityOfResult:** This parameter represents what should be the distance between each checkpoint for the minimization function used in our multilateration algorithm.

**numberOfBlocks, numberOfRooms:** This parameter represents the number of objects that may affect the BLE signals. We know that objects cause some signal loss when an EM signal hits them. Since there could be many obstructions in an indoor environment, we only consider the large objects that my affect the BLE signals as a simplification. Additionally, if rooms exist in an environment, due to having walls around them, they cause some signal loss when an EM signal hits them.

**blockWidths, blockLengths, roomWidths, roomLengths**: *blockWidths* and *blockLengths* represent the widths and lengths of the blocks in the environment in meters, respectively whereas *roomWidths* and *roomLengths* parameters represent the widths and lengths of the rooms in the environment in meters, respectively.

**blockMaterials, roomMaterials:** Besides the width and length of the blocks and rooms, the material used to make these blocks or rooms should be taken into account since different materials have different *electromagnetic* (EM) signal transmission, absorption and reflection values [27]. *blockMaterials* and *roomMaterials* represent the material of the blocks and rooms respectively. Each of these parameters can contain multiple materials to indicate different materials for each block or room.

**materialSignalDisturbanceCoefficients** It represents he signal disturbance coefficient of the materials as key and value pairs like a hash table data structure. Key and value pairs are represented as key:value where keys are materials and values are the signal disturbances for the corresponding material. Signal disturbance here represents how much dBm of the signal is lost for the corresponding material of 1m width.

**pastCoeff:** The name of this parameter is abbreviation for past coefficient. This parameter represents how much of the previous velocity should be used for determining the current predicted velocity of the tag. We use this coefficient parameter to take human motion into account. Since humans go along in a direction for some time, they make a similar movement for most of the time. For the other times, humans stop and turn into another direction. Hence, thinking that tag will make the same movement as the previous motion would be more probable than not.

Past coefficent value changes between 0 and 1. 0 means only consider the current motion. 1 means only consider the previous motion. The values between 0 and 1 indicate how much of the previous motion we should take into account for the magnitude and direction of the current tag motion. This past coefficient value is ignored for the first motion since there is no previous motion before the first one.

**NumberOfParticles:** This parameter represents how many particles we should use for the particle filtering algorithm.

**movingLimit:** This parameter represents how many meters at most the tag should be able to move for *x* and *y* dimensions, at each time step. In this study, since each time step is assumed to be 1 s, this parameter represents the maximum velocity for the tag is in m/s. For example, to track people driving electric cars in an airport, this parameter can be chosen as 20 m/s.

**FPbeaconPos**: An abbreviation for fingerprinting beacon positions. It represents the positions of the beacons we keep in the environment for a while to collect RSSI values for fingerprinting.

**FPcoeff:** An abbreviation for fingerprinting coefficient. It represents a multiplier determining the effect of fingerprinting map in the OASLTIP algorithm. This parameter can take any positive value. The higher this parameter is the larger the fingerprinting map affects the multilateration algorithm results. If the value of this parameter is greater than 1, then fingerprinting results would suppress the other calculations we do for minimization.

**numberOfReceivers:** This parameter represents how many receiver devices should be placed in the map.

**receiverPositions:** This parameter represents the 2D positions of the receivers installed in the IE.

**TXPower:** This parameter is related to beacons. Beacons emit signal with a certain power. This power is represented as milliwatt (mW) normally; yet, in distance calculations, usage of decibel-milliwatts (dBm) is more common. Therefore this parameter represent how much power the beacon transmits the signal in terms of dBm.

**RSSIatOneMeter:** This beacon-related parameter is usually stated in the website of the firm where beacons are bought from. If not stated, one can measure the RSSI value at a receiver which is 1 meter away from a beacon as this value. We choose RSSI at 1 Meter value equal to TXPower−65 since we generally see that this value is (TXPower−65)±3 according to different beacon firms. For TX Power values lower than −20 dBm, like −30 dBm and −40 dBm, RSSI at 1 M is generally less than (TXPower−65)±3. Since it is relatively easy to measure signal power at 1 m, this value is used as a reference to make calculations further than 1 m. For distances closer than 1 m, we calculate the RSSI as an exponential function whose resulting value change between TX Power and RSSI at 1 m between distances 0 m and 1 m respectively.

## 5. Experimental Evaluation

We evaluate the validity of our proposed approach both by performing experiments in our simulated environment using the OASLTool we developed and also by experiments in a real-environment. In the simulated environment, we evaluated the localization performance under different circumstances with small and larger maps, with different signal noise levels, with different numbers of receivers and with different types of obstructions. In the real-world experiments, we both compare the results with the real ground truth value and also, we validate the performance of our simulation tool’s performance. Additionally, we perform behavioral analyses to reveal how BLE signals behave in general.

### 5.1. OASLTool Experiments

In our simulated environment experiments, we use two different environment maps of size 14 m × 11 m (large) and 5 m × 3 m (small). We use six time-steps for the small map and 16 timesteps for the large map for tracking. Each experiment is repeated 20 timesteps with different random initial seeds, we report the mean and the standard deviation of the performance metric. For localization experiments, we report mean absolute error as our performance metric.

(8) $$MAE=\frac{\sum_{i=1}^{T}\left|\right|\left[x_{t},y_{t}\right]-\left[{\hat{x}}_{t},{\hat{y}}_{t}\right]{\left|\right|}^{2}}{T}$$

where *T* is the total number of time steps, $\left[{\hat{x}}_{t},{\hat{y}}_{t}\right]$ is the predicted position of the tag, $\left[x_{t},y_{t}\right]$ is the actual position of the tag for time step *t*.

#### 5.1.1. Localization Performance

For measuring the localization performance, we use two different TX powers and two different sized maps and we also test the system with obstructions. We give the multilateration stage results separately in Table 2 and the particle filtering results are provide in Table 3.

Our results show that multilateration stage performs with almost perfect accuracy when there is no noise or obstruction in the indoor environment, which is almost impossible. However, when the signal noise increases multilateration performance decreases, which is normal since noise gives misdirects the positioning information. Generally, getting in the coverage area of more receivers result in more accuracy as can be seen better in large map cases. Moreover, even though we handle the obstructions in the indoor environments, the obstructions may still decrease the performance since we cannot know exact signal loss due to the obstructions. The particle filtering allows us to have a better localization performance and also control the variances due to the differences in the environmental conditions, such as the signal noise level and the presence of obstructions. Hence, overall OASLTIP pipeline results in a more robust performance under realistic conditions.

#### 5.1.2. Effect of the Number of Receivers

We also search for the effect of the increased number of receivers in a large map with 2 concrete blocks and with 10 dBm noise level. The number of receivers we experimented are 1, 2, 3, 5, 7 and 9. We position the receivers in a way that we reach as much coverage area as possible. The more receiver we have, the more chance we receive a signal from the beacon. More receiver also means possible more distance information to the tag that we need to track.

**Figure 6:**
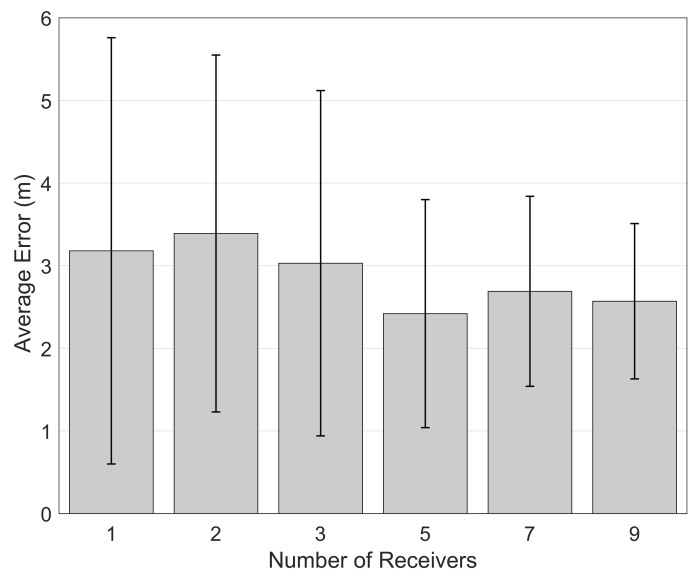
Effect of number of receivers.

We give the results of our experiments in Figure 6. As one would expect, increasing the number of receivers is generally results in better accuracy in general also helps decreasing the variance of the results. It is also important to note that even with a single receiver the performance of the OASLTIP algorithm is not reduced extremely making our solution also a cost-effective one.

#### 5.1.3. Effect of the Signal Noise Level

**Figure 7:**
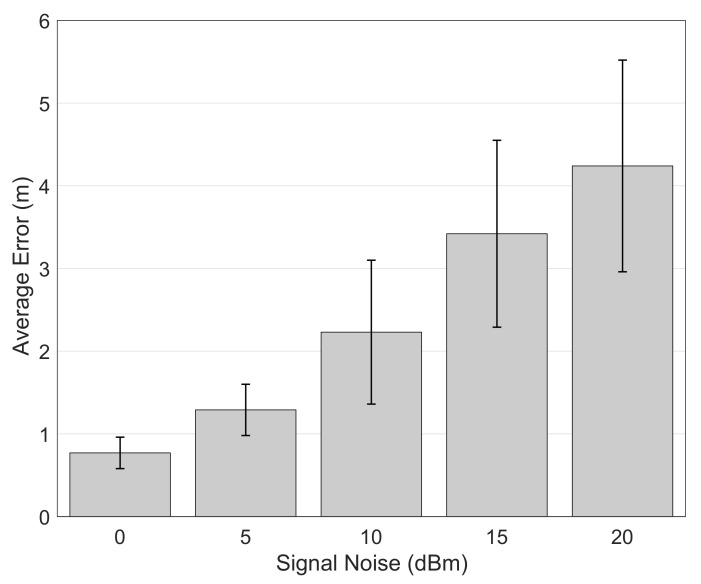
Effect of noise level.

Signal noise is an undesired but unavoidable phenomenon which misdirects positioning calculations. We depict the effect of the signal noise on the localization performance in Figure 7 for 0 dBm (no noise at all) up to 20 dBm. Above this level, the signal quality decreases so much that nearly all the signals are filtered at OASLTIP prefiltering step and tracking becomes almost impossible. As the noise increases, our particle-filter tracking algorithm’s confidence levels drops but it still generates reasonably accurate localization performance due to its ability to remember previous states.

#### 5.1.4. Effects of Obstruction Material Type and Thickness

In order to observe the effects of obstruction material types, we use glass and concrete blocks of 30, 50 and 70 cm width. In order to challenge the method, we use the small map and the number of blocks are chosen as two. The experiment environment and places of the obstructions are shown in Figure 5. The material signal loss information is taken into account in accordance with the values found in different studies [27,28,29]. These values can also be adjusted accordingly since materials may not be in pure form in real world. In our experiments, we use 16 dBm and 6 dBm for concrete and glass signal losses per 1 m thickness.

**Figure 8:**
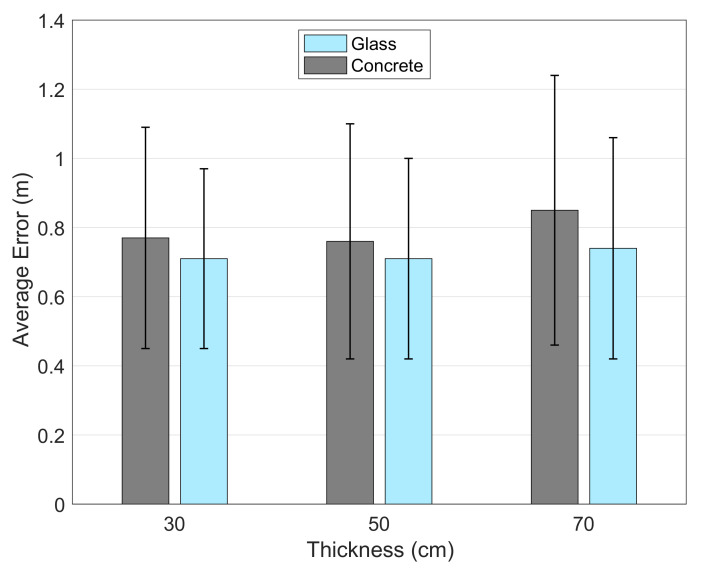
The effect of thickness for each obstruction material.

The results are given in Figure 8. In terms of the material type, concrete causes more signal loss than glass does. As the thickness of the objects increase, the signal loss they cause increases. In both types, our system performs well with increased size obstacles since we consider the losses due to the obstruction material and its thickness. Although, 50 cm blocks cause more signal loss than 30 cm definitely, our algorithm is able to correct the signal according to the obstructions and still provide as accurate location estimation as the 30 cm case. The mean errors are almost identical in both cases but the variances are higher in 50 cm case. That is due to the algorithm’s ability to compensate for the signal loss. When we consider the 70 cm case, for the glass blocks, we can still get a decent accuracy comparable to the other cases, however, the concrete blocks cause more degradation of performance.

### 5.2. Real-World Experiments

In this section, we first describe the experiments that we conduct in a real-world environment to show how OASLTIP algorithm is used and how accurate the results are. After that, we also share our insights on behavioral characteristics of wireless signals.

The second type of experiments are performed to understand how BLE signals behave in general. We call the second type of experiments the behavioral experiments. To come up with a solution using BLE signals, we need to know how BLE signals behave and how these signals should be used in different situations. We search for the effects of signal blocking materials, antenna, chipset and transmission characteristics in these experiments.

#### 5.2.1. Positioning Accuracy Experiments

**Figure 9:**
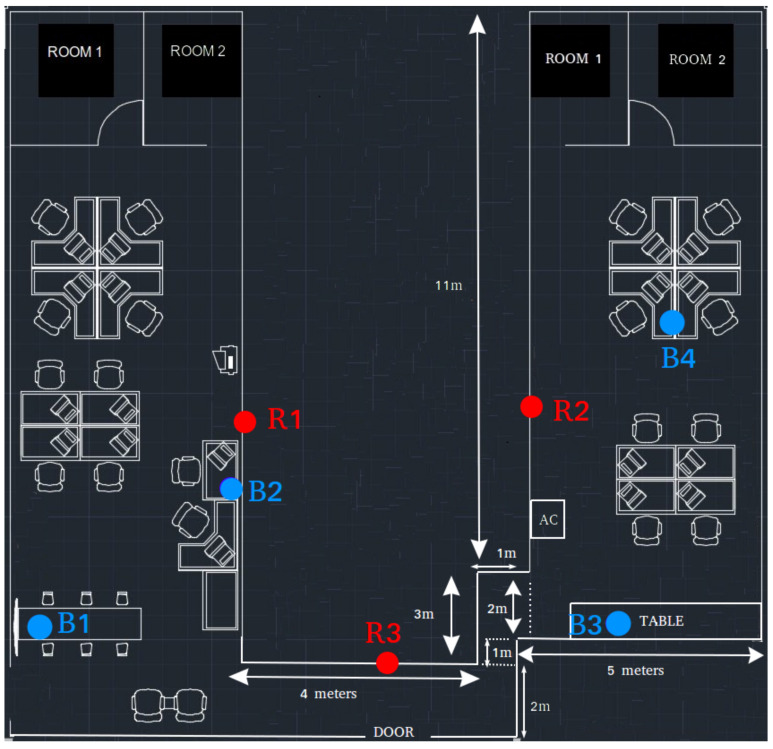
Indoor environment for the real-world localization experiments.

For the positioning experiments, the signals were collected from the receivers we placed on the walls of our test environment. Then, the block and room position information was entered into our indoor positioning system (IPS). We performed the experiments in an office environment; the layout is shown in Figure 9. Blue dots represent beacons and red dots represent receiver devices on the wall. Beacons are prefixed with letter "B," whereas the receivers are prefixed with letter "R" to make them easily distinguishable from each other.

We also needed sizes of blocks and rooms for our simulation, and this information can also be seen in Figure 9. There was a corridor that connected to vertical sections and one of the receivers was placed on the wall in this corridor. The other two receivers were placed in the vertical sections. All receivers were 2 m above the ground and all the beacons approximately 1 m above the ground. The Euclidian distances of the fingerprinting beacons to the receivers are given in the Table 4.

**Figure 10:**
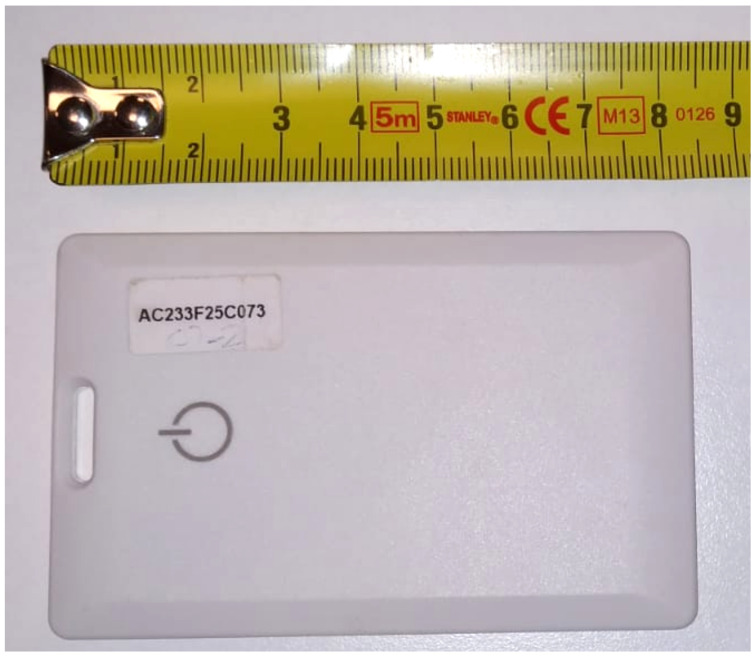
Card beacon.

**Figure 11:**
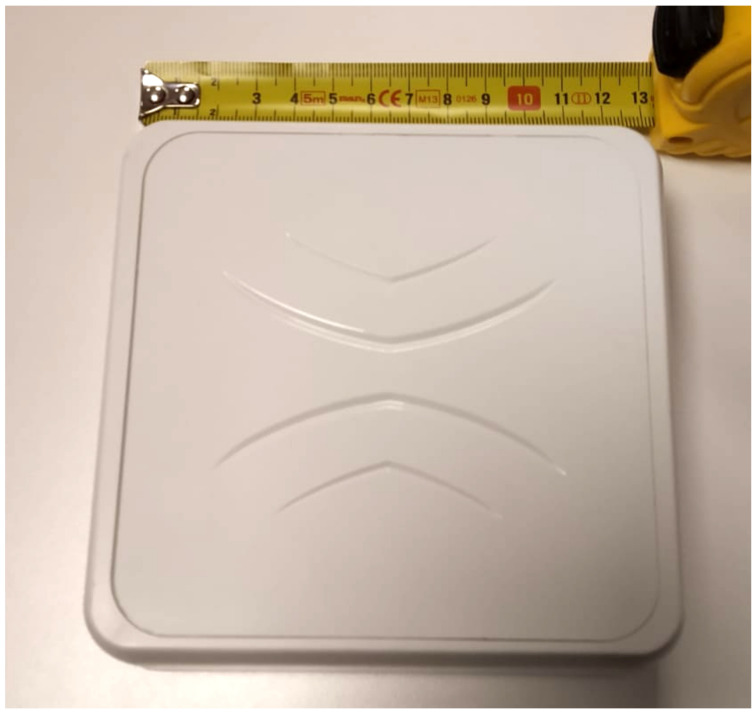
Receiver device.

We used card shaped BLE beacons that use iBeacon technology to emit BLE signals, which is shown in Figure 10. The width, length and thickness of the BLE beacons we used were approximately 86 mm, 55 mm and 4 mm respectively. We used an embedded device with a single-board computer that had a BLE antenna which is shown in Figure 11.

We know the places of the fingerprinting beacons. Therefore, RSSI values of these beacons for different receivers give us some idea about the signal power value and the positions on the map. To construct a fingerprinting map to be used in OASLTIP algorithm, we placed four beacons at certain positions in an office environment and collect signal power results for approximately three days. We called these beacons fingerprinting beacons. We used MACIDs of beacons to identify each tag. The TX powers and advertising intervals of the beacons were 0 dBm and 1000 ms for these measurements.

Real-world experiments were conducted in an office environment. We have three cases where we performed the real-world experiments in three different conditions:

1. In-pocket, crowded (carrying the beacon in our pocket in a crowded environment).
2. In-hand, crowded (carrying the beacon in our hand in a crowded environment).
3. In-pocket, non-crowded (carrying the beacon in our pocket, in a non-crowded hour).

**Figure 12:**
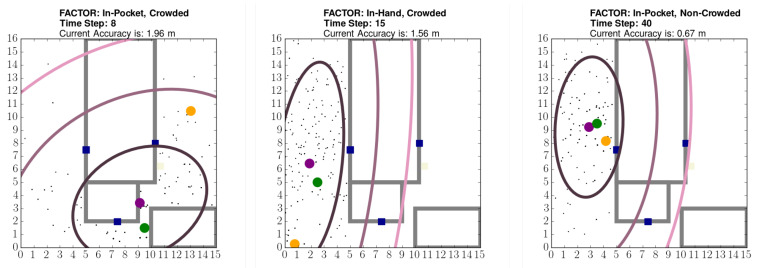
Example screens from the experiments.

In our setup, crowded means that the population density changed between 0.07 people/m^2^ and 0.10 people/m^2^, whereas non-crowded means that the density was 0.3 people/m^2^. We walked at average speeds of 0.41, 0.61 and 0.80 m/s with standard deviations of 0.60, 0.32 and 0.30 m/s for case 1, case 2 and case 3 respectively. Example screens from the OASLTool are given in Figure 12.

For the ground truth position values when moving, we used a fish-eye lens camera results; we used the position values only in sight of the camera. While comparing the OASLTIP localization results with the ground truth positions, an important issue is to match the camera time and the time RSSI values received at all of our receivers. To that end, we used a server to synchronize times for each of our receiver devices and the camera. We made sure that they were perfectly synchronized in terms of seconds. Another issue is we needed to collect some data to understand the signal behavior in the environment. Hence, in this experiment, we collected the signal power values for three days at 10-min time intervals and then averaged them.

We applied our algorithms and methods on our receivers, which are embedded devices that have Bluetooth 4.0 chipsets supporting BLE. Data are acquired via BLE antennas and BLE chipsets that are built into our receiver devices. These BLE chipsets are built-in on a single-board computer chip, but the antennas are replaceable.

To find out how realistic our simulation tool is, we performed two different types of experiments for the positioning performance for the same real-world environment:

1. **Real-World RSSI Data:** These experiments were performed in an office environment with real data. With these experiments, we could determine the performance of the OASLTIP algorithm in real life. We firstly noted down the obstruction information in the office environment. Then, we wandered around the environment for 50 s and then collected the RSSI values at each receiver during the time of wandering. After that, watching the recordings of a fish-eye lens camera, we noted down the ground truth positions for each second. Lastly, before running the OASLTIP algorithm, we entered the obstruction position information, ground truth positions and the collected RSSI information into the algorithm. Using the entered information, our algorithm predicts a position with some confidence and calculates how the predicted position differs from the ground truth positions at each time step.
2. **Generated RSSI Data:** These experiments were also performed in an office environment with real data except for the RSSI data. These experiments were designed to show how realistic our simulation tool is in terms of generating RSSI data for a real-life case. We applied the same procedure as in "Real-World Data Experiments" except for the RSSI collecting part. In these experiments, we used RSSI data that our simulation tool generated by looking at the ground truth positions resulted from wandering around the office and obstruction information in the real-world environment.

In both types of experiment, we used BLE fingerprinting data that we collected for three days in the real-world environment.

We report the mean absolute error (MAE) results together with the number of no signal reception cases in Table 5. According to the results, we observe that we are able to reflect the effects of large objects and rooms in our results. However, in the real world, there are also the humans and small objects in the environment which dynamically change. Our algorithm takes static objects residing in the environment into account and tries to mitigate the effects of these objects for positioning calculations.

**Figure 13:**
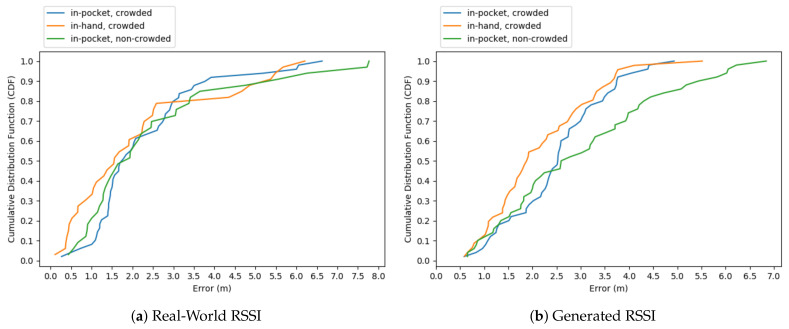
Cumulative distribution of errors.

We also give our results using the cumulative distribution function of errors in Figure 13. Our method guarantees an error lower than about 2.5–3.5 m in 80% of cases depending on the scenario with the real-world data. Once we generated the RSSI values using the simulation tool, we could see that, for the crowded cases, we achieved less than 3.5 m, and for the non-crowded case, we achieved less than 4.5 m at 80%.

Our real-world office environment had a difficult architectural plan for tracking with a small number of receivers. That is why our errors can go as high as 8 m, only momentarily, when the tag reaches at the corners of our U-shaped office environment. For localization, normally, at least three receivers are needed for distance calculations based on RSSI. However, our algorithm always tries to find a position even when we have one RSSI value at only one receiver. We prevent doing calculations when none of our receivers has RSSI values in the real-world experiment, since it could mean the person went out of the indoor environment. However, if we know that the tag is always in the environment, then the OASLTIP algorithm can make positioning calculations even without receiving a single RSSI value, since not receiving any signal may be interpreted as being a certain distance away from the receivers.

#### 5.2.2. Behavioral Observations

In order to provide insights about the signal behavior that we encountered during our real-world experiments, we provide some tests on the physical characteristics.

**Signal Blocking Materials:** We used two 2.4 GHz Wi-Fi dongles in this experiment, in which one of them is positioned on the right and the other was positioned on the left with 20 cm space between them. We put two aluminum barriers having thicknesses of 0.5 cm between the two dongles; each barrier covered top, bottom and left positions for the right dongle and top, bottom and right positions for the left dongle. We used Wi-Fi signals for this test, and the results were meant to be similar to the results below for BLE signals, except for the fact that BLE signals are be lower in strength. We used aluminum since aluminum is known to block high frequency electromagnetic signals like Bluetooth well [29]. We performed this test while being approximately 50 cm away from both of the dongles, starting at the right dongle which was positioned at $0^{\circ }$.

**Figure 14:**
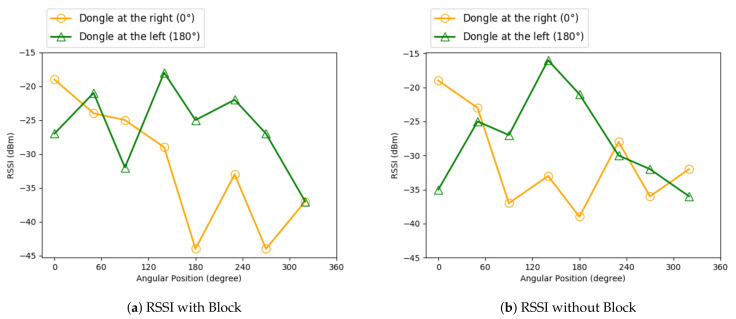
RSSI comparisons.

In Figure 14, we can see the RSSI results for a beacon that gets hold in different angles and positions to the dongles (receiver devices) to see the effect of material just near the receiver devices. Having a block in between decreases the RSSI for the dongle at the left, preventing the beacon and the left dongle from being in line of sight with each other. However, at $100^{\circ }$, somehow, the right dongle has a higher RSSI value in the case with blocks than in the case without a block. Hence, we can say that the positions of the beacon and the receiver devices and the angle between them are also factors that need to be taken into account besides the presence of an obstruction alone. To receive stronger signals, we should place the receivers in positions and angles such that their BLE chipset is in sight with the surrounding BLE signals as much as possible.

**Antenna:** We take RSSI measurements of a beacon at a receiver with and without an antenna. The beacon we used has 0 dBm as TX power, and therefore, has an expected RSSI of approximately −70 dBm at 3 m. As a result, we observed that the antenna plays a huge role in receiving the RSSI values. The receiver got RSSI values of approximately −70 dBm when we were 3 m away from the receiver and got signals after 15 m easily. However, after removing the antenna, the RSSI values dropped to −93 dBm for 3 m and we could not get any signal beyond 7 m. An antenna is a must-have for receiver devices. Alternatively, we can use receiver devices when the antenna is removed to check if a beacon is near because if these types of receivers receive a signal, then it must be due to a nearby beacon. In this way, we eliminate the need for checking RSSI values for near proximities.

**BLE Chipset/Receiver Device:** We tested different receivers with two different well-known and quality chipsets. We wandered around an office environment and checked the RSSI values at the receiver devices having different chipsets to see whether the chipset affects the RSSIs. According to our observations, chipsets also affect the signal reception, since one of our receiver devices catches signals that the other one cannot, most of the time. BLE chipset or receiver device quality is an important factor for accurate positioning and one should buy a quality receiver device.

**Obstruction Effect:** For these tests, we held the beacons when there was a 30 cm wall completely blocking the way between the receiver and the beacons. Then, we tested when there was no wall between the receiver and the beacon to see the effect of the wall for BLE signal penetration. As a result, one of the receivers was able to receive signals from a beacon beyond a wall while the other one was not. This shows that the signal gets severely degraded due to the wall, and with a receiver having a weak chipset, it is very hard to obtain any signals when there is a thick obstruction between the receiver and the beacons. According to the results, we can say that the receivers should be placed in a way that the obstructions between the receivers and the beacons are minimal.

**Transmitter/Beacon:** We tested several beacons from different firms, but after some tests for decision making, we chose three of them each belonging to a different firm. The beacons we used in this study were (i) C7 from Minew [30]; (ii) H3 Card beacon from Moko smart [31]; and (iii) Card Tag from kontakt.io [32]. The first two beacons have Bluetooth 5.0 and the third one has Bluetooth 4.0. All beacons have 0 dBm transmission power and RSSI at 1 m are all −65 dBm. All of the beacons use iBeacon protocol. The sizes of the beacons are about the same. Since carrying in hand results in greater achievable distances, we can see that human body and the clothes between the receiver and the beacon decreases the signal power. As for Bluetooth versions of beacons, the higher the version of Bluetooth, the better the beacon is if the receivers are compatible with the versions of the Bluetooth. As can be seen from the results, if receivers do not have as high Bluetooth version as the beacons, then having a higher versioned beacon may not be necessary.

**Transmission Power and Period:** In total, we investigated eight different TX powers, which were −40, −30, −20, −16, −12, −8, −4, 0 dBm; and four transmission periods, which were 100, 300, 800 and 1000 ms. According to our experiments, we observed that increasing TX power increases the signal stability and provides more healthy decisions for positioning. However, as the transmission period decreases, the battery consumption increases which is a trade-off that one should consider. According to our observations in general, BLE signals having a ≤300 ms advertising interval (transmission period) results in a stable signal environment for signal positioning calculations. We chose 0 dBm TX power and a 1000 ms advertising interval for real-world experiments. Since the advertising interval we choose was lower than 300 ms, our signal was not very stable. However, it is necessary to make the battery last longer. We did not decrease the TX power to have a higher battery life since low TX powers fade away easily in noisy or large environments, which was against our aim to be able to track the tag in large and noisy environments. As expected, as the TX power increased or the advertisement period decreased, the stability of the signal increased, thereby improving the indoor positioning accuracy.

### 5.3. Discussion

We summarize our findings from the experiments in this section. In localization experiments, since our beacons do not transmit BLE signals frequently, all of the receivers may not catch signals at each time step which may lower the accuracy of our multilateration algorithm. Therefore, we determine a way to use signals for the time steps we do not receive any. To that end, we firstly search for the previous time step’s RSSI value and if not found, then wait for the next time step’s RSSI value. We use a parameter to determine the maximum value for how many time steps into the past or into the future we should look at until we find a RSSI value. For our real-world test, we search for at maximum two past and two future values. If we do not find any signal, then we mark the signal value as none. In this approach, our real time algorithm may make the current time step’s calculations after up to two time-steps (2 s in our case) later to find a signal. Since this approach may use the same signal multiple times, our sliding window size requirement for the prefiltering step of OASLTIP should be increased to be more selective and hence, we set the minimum size requirement of each sliding window to five instead of three.

We realize that when carrying beacons in hand or in pocket, movement affects the signal powers. Therefore, we add 4 dBm to RSSIs before making the calculations for the cases carrying in hand and add 6 dBm for the cases when we carry the beacons in our pockets. We expected carrying in hand would be much more accurate than carrying in pocket factor, but after adding these offset power values, results did not change much in our case. We also observe that moving beacons decreases the RSSI values at receivers compared to keeping them still.

According to our observations, BLE signals having ≤300 ms advertising interval results in a stable signal environment. By stable signal environment, we mean an environment where receivers can get enough signals to update the positioning result more often to catch up with the tag motion. However, since we use 1000 ms as the BLE signal advertising interval, we often lose the signal at our receivers which prevents making positioning calculations more often. Additionally, we use low number of receivers for a large office environment where there is no clear line of sight between any of our receivers and there are lots of wireless devices like cell phones and Wi-Fi access points interfering with the Bluetooth signal. However, since we can handle cases where we receive no signal at a receiver, our algorithm can still achieve accuracy similar to the related studies.

We also use fingerprinting information in our multilateration algorithm to find the best matching position given RSSIs. Even though, our fingerprinting results do not seem consistent with formulas for conversions between RSSI and distance, they naturally take environmental factors into account which helps us make more accurate positioning predictions. In our case, fingerprinting does not always increase the accuracy for some positions and sometimes decreases it. However, if we have more beacons positioned for fingerprinting, we might get better results for these positions as well.

The results show us that the simulation tool is more optimistic about the results than it should be in reality. Furthermore, the simulation tool generally assumes to receive a signal if the beacons are in the range, yet, we see that, in real world, the receivers do not get signals all the time even if the receivers are in the range of the beacons according to the transmission powers of the beacons.

Even though the localization performance in *Real-World RSSI Data Experiments* is not enough for strict positioning applications which require sub-meter accuracy, they are accurate enough to show which part in a room the tag is close to. Our performance meets our need to have a system with long battery life and low hardware costs with decent localization accuracy.

Our method cannot handle multiple floors currently, but different floors can be integrated as different environments in the simulation tool. Additionally, currently, we assume that BLE signals propagate linearly. However, in real life, BLE signals do not propagate linearly and they get affected from multipath effect. Hence, in further studies, more suitable propagation models for BLE signals could be used.

## 6. Conclusions

In this study, we presented the OASLTIP algorithm to perform indoor localization and tracking using BLE tags. OASLTIP has a pipeline which consists of pre-filtering, multilateration and particle filtering-based tracking steps. Unlike many of the existing studies, our algorithm does not assume the existence of fingerprinting information, and it can work with less than three receivers. Additionally, it can handle a signal loss of up to several time-steps. These are the features that makes our approach very robust and suitable for diverse and challenging setups. Besides, in order to make the location measurements more precise, our method takes into account the signal losses incurred by the obstructions that exist in the environment. OASLTIP does not report only the measurement result but also provides an additional tracking stage that uses a particle filtering algorithm to incorporate the previous movement of the tag. This yields a better, smoother and consistent estimate of the position.

There are three main stages in the OASLTIP method: (i) prefiltering, (ii) multilateration and (iii) tracking using particle filtering. The prefiltering step smooths the new RSSI reading using average filtering and also discards the signal if the quality is low. Since we discard low-quality signals, in the multilateration step, we tolerate the potential loss of signal. We impute it with the average of recent previous measurements that have higher quality. Additionally, in multilateration, we take into account the architectural constraints and obstructions in the environment. We consider the signal propagation losses due to obstructions by approximating the loss using the thickness and the type of material that is in between the receiver and the beacon. In general, obstruction information is considered using indirect methods like fingerprinting. In our approach, we integrate this information into the core of the positioning algorithm itself and leave the fingerprinting as an optional step.

We performed several experiments to validate our approach under difficult conditions in both simulated and real environments. We also validated the performance of our simulated environment by generating the real-world configuration in our flexible simulation tool and repeating the real-world experiments. This way, we have more information about the approximation capacity of our simulation tool compared to real-world scenarios. To our knowledge, this is the only study that adopts such an approach. Finally, we provided our insights from the experiments on the behavior of BLE signals in terms of different chipsets, different transport positions, different transmit powers and different obstructing materials.

We are releasing our experimentation framework tool along with our algorithm. OASLTool is capable of simulating, visualizing and reporting the results for diverse scenarios that can be configured in a flexible manner. OASLTool also visualizes how much we are sure about the positioning result using confidence ellipses formed around the tag position. We showed different simulations for different scenarios to show the theoretical performance for different cases. We also performed real-world experiments in an office environment which was 15 m × 16 m. As a result of 50 s of wandering around the environment, we achieved an average error of 2.29 m with a standard deviation of 1.67 m.

There are different methods and approaches using the BLE technology in the literature. We see that some of these methods achieve lower errors than our method. However, those approaches generally rely on frequent (<1000 ms) and high-powered signals transmitted from beacons or frequent receiver placement in the indoor environment. In this paper, we proposed a cost-effective and robust algorithm which does not rely on frequently installed receivers or frequently transmitting beacons. Obstruction information in the environment is integrated into the core of the algorithm.

This study can be further extended with different transmission power and advertisement interval values. Moreover, in this paper we assumed that BLE signals propagate linearly. In further studies, in terms of collision detection of signals with obstructions, a suitable propagation model for BLE signals can be taken into account as well.

## Figures and Tables

**Table 1:** BLE indoor positioning-related studies.

MAE (m)	Experiment Area (m)	Deployment Requirements	Method and Reference
0.192	5×5	3 beacons	Kalman/Gaussian filter, trilateration-centroid [10]
0.76	5.6×8.8	8 fixed beacons	Hybrid, sliding window + KF, trilateration, DR [15]
0.8	10.5×15.6	22 fixed beacons	BLE fingerprinting [22]
1.0 with 75%	8×8	4 beacons, 5 Wi-Fi AP	Hybrid, SVM, KNN, ML cloud service [13]
1.27	90×37	48 fixed beacons	Graph optimization, fingerprinting+range based [23]
1.37	8.4×15	BLE tags and 4 Repeater	Hybrid (Wi − Fi + BLE), fingerprinting, CoO [14]
$\sqrt{2}$ with 94%	30 m^2^	4 fixed beacons	Selection of nearest BLE tag [16]
1.5	6×6	9 fixed beacons	Trilateration, RSSI filtering [24]
μ:2.29, σ:1.67	15×16	1 beacon, 3 receivers	OASLTIP
$2\sqrt{2}$ with 70.2%	5×5	4 fixed beacons, 1 receiver	Two machine learning classifiers [20]
2.89	12×14	4 fixed beacons	Trilateration, min-max, least square [9]
4.0 with 97.5%	8×6	5 beacons	Particle filter, BLE fingerprinting [18]
4.6 with 90%	16.5×17.6	4 receivers, 1 beacon	Channel diversity, weighted trilateration and KF [21]
1.5 with 91.4%	3 (1D)	1 beacon	Low TX powered beacon utilization [17]
0.5	3 (1D)	1 beacon	IPSAPP (A mobile application) [11]

**Table 2:** Multilateration location prediction errors in meters.

	Small Map		Large Map	
**Noise Level**	**0 dBm**	**10 dBm**	**0 dBm**	**10 dBm**
No Obstruction	0.06 (σ=0.01)	1.06 (σ=0.46)	0.04 (σ≃0.00)	2.26 (σ=0.43)
2 concrete blocks	0.67 (σ=0.50)	1.00 (σ=0.45)	1.80 (σ=0.98)	2.66 (σ=0.98)

**Table 3:** Particle filtered location prediction errors in meters.

	Small Map		Large Map	
**Noise Level**	**0 dBm**	**10 dBm**	**0 dBm**	**10 dBm**
No Obstruction	0.52 (σ=0.15)	0.77 (σ=0.21)	0.77 (σ=0.22)	2.17 (σ=0.77)
2 concrete blocks	0.83 (σ=0.32)	0.71 (σ=0.27)	1.91 (σ=0.86)	2.33 (σ=0.89)

**Table 4:** Receiver-fingerprinting beacon distance (m).

	Receiver 1	Receiver 2	Receiver 3
**Beacon 1**	7.08	11.58	7.15
**Beacon 2**	1.5	5.66	4.66
**Beacon 3**	7.64	4.66	4.37
**Beacon 4**	7.65	2.42	8.66

**Table 5:** Real- world experiment—location prediction performance.

	Real-World RSSI		Generated RSSI	
**Metrics**	**MAE in m**	**#No Signal**	**MAE in m**	**#No Signal**
Case 1	2.28 (σ=1.39)	5	2.50 (σ=1.00)	4
Case 2	2.13 (σ=1.77)	17	2.17 (σ=1.08)	4
Case 3	2.47 (σ=1.92)	21	3.01 (σ=1.66)	4
Combined	2.29 (σ=1.67)	43	2.58 (σ=1.33)	12

## Data Availability

The data and the code presented in this study are openly available in https://github.com/aybarskerem/OASLTIP.
